# Epidermal growth factor receptor T790M mutations in non-small cell lung cancer (NSCLC) of Yunnan in southwestern China

**DOI:** 10.1038/s41598-018-33816-x

**Published:** 2018-10-18

**Authors:** Yongchun Zhou, Yuhui Ma, Hutao Shi, Yaxi Du, Yunchao Huang

**Affiliations:** 1 Cancer Research Institute of Yunnan Province, The Third Affiliated Hospital of Kunming Medical University (Yunnan cancer Hospital), Kunming, 650118 P.R. China; 2 Department of Thoracic Surgery I, The Third Affiliated Hospital of Kunming Medical University (Yunnan cancer Hospital), Kunming, 650118 P.R. China; 3 Department of imaging, The Kunming Tongren hospital, Kunming, 650118 P.R. China; 4 Key Laboratory of Lung Cancer Research of Yunnan Province, The Third Affiliated Hospital of Kunming Medical University, Kunming, 650118 P.R. China; 5 International Joint Laboratory on High Altitude Regional Cancer of Yunnan Province, The Third Affiliated Hospital of Kunming Medical University, Kunming, 650118 P.R. China

## Abstract

To explore the effect of epidermal growth factor receptor (EGFR) T790M mutation status on non-small cell lung cancer (NSCLC) in Yunnan province of southwestern China. First, this study used the super amplification refractory mutation system (Super ARMS) polymerase chain reaction (PCR) and Droplet Digital PCR (dd PCR) to evaluate the T790M gene mutation, in plasmatic ctDNA samples from 212 cases of NSCLC. The association between T790M mutations and clinical parameters were further explored. Next, to investigate the mechanism of drug resistance that resulted from T790M mutation, subgroup analyses according to duration of medicine (EGFR-TKIs) were carried out. Finally, we also evaluate the effectiveness of blood-based circulating tumor DNA (ctDNA) on detecting the T790M mutation by calculating Super ARMS’s detection efficiency. We found that the T790M mutation rate was 8.4% (18/212) in overall patients. The T790M mutation was more frequent in patients with brain metastasis 30.0% (12/40) (*p* < 0.01). We found that post-TKI samples 42.8% (15/35) were associated with a higher T790M mutation rate (*p* < 0.01). Subgroup analysis showed that the duration of TKI therapy for 6 to 10 months 66.6% (8/12) (*p* < 0.01) and >10 months 75.0% (9/12) (*p* < 0.01) were also associated with a higher T790M mutation rate. Super ARMS’s sensitivity, specificity, PPV, NPV, and accuracy were 100.0%, 99.4%, 94.7%, 100.0%, and 99.5% respectively. Generally, the EGFR-T790M mutation was more common in NSCLC patients with brain metastasis and those who received TKI therapy for more than 6 months. Moreover, Super ARMS is a sensitive, efficient, and practical clinic method for dynamically monitoring T790M mutation status and effectively guiding clinic treatment.

## Introduction

Lung cancer is the most malignant tumors with the fastest growth rate of morbidity and mortality in the worldwide. In China, the peak of morbidity and mortality has never fallen. According to the statistics, the number of new cases of lung cancer is about 326,600 and the death number due to lung cancer is about 569,400 in 2012^1^. Additionally, NSCLC accounts for 85% of all lung cancer cases^2^.

Xuanwei City, in the northeastern part of Yunnan province, China, is 6,257 km^2^ and lies on high plateau punctuated by mountain ridges^3^. Its total population is approximately one million. More than 40% of the males, but less than 0.1% of females, smoke tobacco. Although there are some local industries, including coal mining, electric power generation, and light manufacturing, over 90% of the residents are famers. Therefore, we do not consider industrial pollution as a major factor in causing lung cancer. Instead, the characteristics of lung cancer in Xuanwei city were as follows: (1) a relatively high incidence and mortality rate of lung cancer in women and almost all of them did not smoke; (2) the major type of lung cancer in females was adenocarcinoma^4,5^. Previous studies indicated that the lung cancer rate of Xuanwei City of Yunnan Province is higher than elsewhere in China. The primarily because of high incidence and mortality of lung cancer were exposure to indoor air pollution from smoky coal emissions that contain high levels of polycyclic aromatic hydrocarbons (PAHs). The connection between the exposure and lung cancer risk might be increased by a GSTMI-null genotype as well as overexertion of the p53 protein^6,7^. Even after a stove improvement project was done in the late 1980s, although the mortality rate of some regions in Xuanwei started to decline, total mortality rate was still high or even elevated^4^. Based on this result, we speculate that, apart from environment factors, genetic factors may also play an important role in the development of lung cancer in Xuanwu City. Our previous studies found the rate of EGFR mutations was different from that of the general population (higher G719X and G719X + S768I, but lower 19 deletion and L858 mutations) in Xuanwei City^3^. Therefore, whether the EGFR-T790M mutation has regional specificity in Xuanwei has become a main health problem that attracts our attention.

EGFR, a 170-kDa (1186 amino acid) membrane-bound protein encoded by 28 exons spanning nearly 190,000 nucleotides on chromosome 7p12, is one member of the EGFR-TK family, which belongs to a subfamily of four closely related receptors: HER-1/ErbB1, HER-2/neu/ErbB2, HER-3/ErbB3, and HER-4/ErbB4. EGFR-activated pathways include Akt and signal transducer and activator of transcription (STAT) cascades, which are important for cell survival, and the mitogen-activated protein kinase (MAPK) pathway, which induces proliferation^8^. There are four key discoveries about EGFR. First, EGFR was found to be a protein TK involved in cellular signaling^9^. Second, EGFR was shown to be an oncogene, capable of inducing cancer when aberrant^10^. Third, EGFR was shown to be expressed in multiple cancer types at elevated levels relative to normal tissues^11^. Finally, investigators demonstrated that the use of specific monoclonal antibodies against EGFR could inhibit its activity^12^. Platinum-based chemotherapy is the first-line anti-tumor drug for NSCLC^13^. Due to EGFR’s association with malignancies, it has become the target for an expanding class of anti-cancer therapies, such as gefitinib (Iressa) and erlotinib (Tarceva), which are the first-generation EGFR-TKIs. Additionally, TKI therapy may achieve better objective remission rate (ORR) and longer progression free survival(PFS) for patients harboring active EGFR mutations^14^. National Comprehensive Cancer Network (NCCN) guidelines was strongly recommend EGFR testing in NSCLC, and TKIs are also recommended as a first line treatment for NSCLC patients with sensitive EGFR mutations^15^.

Although lung cancer patients who experienced rapid, durable, complete or partial responses to TKI therapy have been found to harbor somatic mutations in the EGFR gene^16^, it has also been demonstrated that many of the patients who had received first generation of TKI treatment for 6 to 10 months will develop resistance to TKIS. The T790M mutation in exon 20 occurs in ~60% of EGFR-mutated lung cancers that have developed acquired resistance to TKI therapy^17^. Therefore, it is important to predict the curative effect by monitoring T790M mutation status.

Currently, NSCLC patients’ tumor tissues are the gold standard for the measure of T790M mutations, and which are usually obtained by biopsy or surgery. However, when diagnosed to be suffering from NSCLC, more than half NSCLC patients (70%) have already been in the advanced stage, and some have lost the precious opportunity of surgery. Meanwhile, repetition of a biopsy is not feasible and will increase discomfort for those NSCLC patients with recurrent disease or acquired resistance to TKIs. Therefore, noninvasive detection of T790M in plasma circulating tumor DNA (ctDNA) has been proved to be feasible as re-biopsy of tumor tissue. It can provide the same genetic information as a tissue biopsy and can be performed at any time during the course of therapy, allowing for dynamic monitoring of molecular changes^18^. Recently, AZD9291 has been listed in the United States. It is an oral, potent, irreversible TKI, that is selective for TKI–sensitizing mutations and the T790M resistance mutation. Therefore, continuously monitoring T790M mutations dynamics in plasma ctDNA could predict the clinical outcome of TKI (AZD9291) and guide further therapy for advanced NSCLC patients. The Super ARMS, used in our study is a reinforced technology based on traditional ARMS.

Since T790M mutation testing facilities (only plasmatic ctDNA) had been established in our center in 2013, T790M mutation testing was performed in local patients with NSCLC to provide the guiding information. As T790M mutation status in NSCLC patients has not yet been comprehensively reported in Yunnan province, the aims of this study were designed to reveal the T790M mutations profile of NSCLC patients in Xuanwei City and to further investigate the significance of T790M detection for NSCLC patients with acquired resistance to TKIs and to further effectively guide clinical treatment.

## Results

### Baseline characteristics of included patients

In this study, two hundred and twelve NSCLC patients were selected by pathologically analysis. These NSCLC patients were consisting of 81 females and 131 males. The mean age was 57.1 ± 11.2, and ranging from 31 to 86 years old. The most common histology was adenocarcinoma 173 (81.6%), and the remaining were squamous carcinoma 21 (9.9%), or another kind of NSCLC were 18 (8.4%). Additionally, 73.5% of the patients were diagnosed as stage IV. 95 (44.8%) patients were smokers, and the remaining 117 (55.1%) were never-smokers. 11 (5.1%) patients had a family history of malignancy. 20 (9.4%) patients were from Xuanwei City. 35 (16.5%) patients had a history of TKI treatment, 61 (28.7%) patients had a history of chemotherapy treatment, 29 (13.6%) had a history of other treatment and the remaining 87 (38.2%) had never received any treatment. Table 1 summarized the main baseline characteristics of included patients.

**Table 1:** Patient characteristics.

Factors	No. (%)
Age (Years)	
Median	57.1
Range	31–86
Gender	
Male	131 (61.7)
Female	81 (38.2)
Pathology	
Adenocarcinoma	173 (81.6)
Squamous	21 (9.9)
Other	18 (8.4)
Tumor Stage	
IA	7 (3.3)
IB	7 (3.3)
IIA	5 (2.3)
IIB	2 (0.9)
IIIA	10 (4.7)
IIIB	25 (11.7)
IV	156 (73.5)
Smoking	
Yes	95 (44.8)
No	117 (55.1)
Family history of malignancy	
Yes	11 (5.1)
No	201 (94.8)
Brain metastasis	
Yes	40 (18.8)
No	172 (81.1)
Xuanwei origin	
Yes	20 (9.4)
No	192 (90.5)
Immediate prior treatment	
First EGFR-TKI	35 (16.5)
Chemotherapy	61 (28.7)
Other	29 (13.6)
No	87 (38.2)
EGFR mutation-positive	64 (30.1)
EGFR T790M mutation-positive	18 (8.4)

### The incidence of the T790M mutation and its relationship with clinicopathological characteristics

The T790M mutation was identified in 8.4% (18/212) of plasmatic samples (Table 1). The difference rate of T790M mutations were according to whether brain metastasis had happened. It seemed that patients who had brain metastasis (*p* < 0.01) were more likely to have the T790M mutation. However, there was no significantly relationship with patients’ age (*p* = 0.99), sex (*p* = 0.28), histology (*p* = 0.24), smoking (*p* = 0.30), family history of malignancy (*p* = 0.62), tumor node metastasis (TNM) (*p* = 0.13), or Xuanwei origin (*p* = 0.86) (Table 2).

**Tablee 2:** Frequency of EGFR mutation according to clinical characteristics in overall patients.

	Sensitizing mutations				total	*P*	Resistance mutations			total	*P*	Combination of sensitizing and resistance mutations			total	*P*	EGFR mutations		EGFR T790M mutations	
	G719X	19-del	L858R	19-del, L858R			T790M	S768I	20-insertion			19-del, T790M	G719X, S768I	L858R,T790M			N	*P*	N	*P*
	N	N	N	N			N	N	N			N	N	N						
**Age**						0.13					0.38					0.23		0.16		0.99
<65	0	14	7	0	21		7	4	1	12		4	3	2	9		42		13	
≥65	2	4	6	1	13		2	0	0	2		2	4	1	7		22		5	
**Sex**						<0.01					0.71					0.95		0.02		0.28
Male	1	10	3	0	14		5	2	1	8		2	6	2	10		32		9	
Female	1	8	10	1	20		4	2	0	6		4	1	1	6		32		9	
**Histology**						0.03					0.44					0.76		<0.01		0.24
Adenocarcinoma	1	18	12	1	32		9	3	1	13		5	6	3	14		59		17	
Squamous	0	0	0	0	0		0	0	0	0		0	0	0	0		0		0	
Other	1	0	1	0	2		0	1	0	1		1	1	0	2		5		1	
**Smoking**						0.40					0.47					0.33		0.61		0.30
Yes	1	8	4	0	13		3	1	1	5		1	6	2	9		27		6	
No	1	10	9	1	21		6	3	0	9		5	1	1	7		37		12	
**Family history of malignancy**						0.14					0.77					0.69		0.42		0.62
Yes	0	1	3	0	4		0	0	0	0		0	0	1	1		5		1	
No	2	17	10	1	30		9	4	1	14		6	7	2	15		59		17	
**Stage**						0.03					0.66					0.39		<0.01		0.13
Ia	0	0	0	0	0		0	0	0	0		0	0	0	0		0		0	
Ib	0	0	0	0	0		0	0	0	0		0	0	0	0		0		0	
IIa	0	0	0	0	0		0	1	0	1		0	0	0	0		1		0	
IIb	0	0	0	0	0		0	0	0	0		0	0	0	0		0		0	
IIIa	0	1	0	0	1		0	0	0	0		0	4	0	4		5		0	
IIIb	0	6	0	0	6		0	0	0	0		1	0	0	1		7		1	
IV	2	11	13	1	27		9	3	1	13		5	3	3	11		51		17	
**Brain metastasis**						0.21					<0.01					<0.01		<0.01		<0.01
Yes	0	4	4	1	9		7	2	0	9		4	4	1	9		27		12	
No	2	14	9	0	25		2	2	1	5		2	3	2	7		37		6	
**Xuanwei origin**						0.85					0.26					0.99		0.31		0.86
Yes	1	3	0	0	4		1	2	0	3		0	1	0	1		8		1	
No	1	15	13	1	30		8	2	1	11		6	6	3	15		56		17	
**Immediate prior treatment**						0.18					<0.01					<0.01		<0.01		<0.01
First EGFR-TKI	0	1	2	0	3		7	2	1	10		5	4	3	12		25		15	
Chemotherapy	0	5	4	1	10		2	1	0	3		0	1	0	1		14		2	
Other	0	1	0	0	1		0	0	0	0		0	0	0	0		1		0	
No	2	11	7	0	20		0	1	0	1		1	2	0	3		24		1	
**Total**	2	18	13	1	34		9	4	1	14		6	7	3	16		64		18	

### The incidence of EGFR mutations and its relationship with clinicopathological characteristics

EGFR mutations were identified in 30.1% (64/212) of plasmatic samples (Table 1). The differences rate of EGFR mutations were associated with patients’ sex (*p* = 0.02), adenocarcinoma (*p* < 0.01), TNM stage (*p* < 0.01), and brain metastasis (*p* < 0.01). It seemed that female patients who had IIIb-IV stage adenocarcinoma and brain metastasis were associated with high likelihood of EGFR mutations. However, there was no significantly relationship with patients’ age (*p* = 0.16), smoking (*p* = 0.61), family history of malignancy (*p* = 0.42), or Xuanwei origin (*p* = 0.31) (Table 2).

### Detection efficiency of Super ARMS for the T790M mutation

The T790M mutation rate was identified in 8.4% (18/212) of patients by Super ARMS. One sample result was not in agreement with the digital PCR result, so it was judged as a false positive (Table 3).

**Table 3:** Comparisons between Super ARMS detection of T790M mutation status and digital PCR validation results.

Super ARMS	(+)	(−)	Total	Mutation rate
**Droplet Digital PCR**				
(+)	18	1	19	
(−)	0	193	193	
Total	18	194		8.4%

Additionally, the Super ARMS’s sensitivity, specificity, PPV, NPV and accuracy were 100.0%, 99.4%, 94.7%, 100.0% and 99.5%, respectively (Table 4).

**Table 4:** Detection efficiency of Super ARMS for the T790M mutation.

Project	super AMRS
**The results of T790M mutation**	
Sensitivity	100.0%
Specificity	99.4%
PPV	94.7%
NPV	100.0%
Accuracy	99.5%

Note: PPV, positive predictive value; NPV, negative predictive value.

### The relationship between TKI therapy and T790M mutation

The T790M mutation rate was identified in 1.6% (3/177) of plasmatic samples from patients who did not receive TKI treatment. It is much lower than the T790M mutation rate that was identified as 42.8% (15/35) in plasmatic samples from patients who received treatment of first generation TKI therapy (*p* < 0.01) (Table 5).

**Table 5:** Mutation status of T790M in NSCLC samples with a history of first generation TKIs drug used by Super ARMS method.

Taking TKI drugs	Cases number	Number of mutant cases (%)	P
Yes	35	15 (42.8%)	<0.01
No	177	3 (1.6%)	

### Incidence of the T790M mutation in duration of TKI subgroups

**Figure 1:**
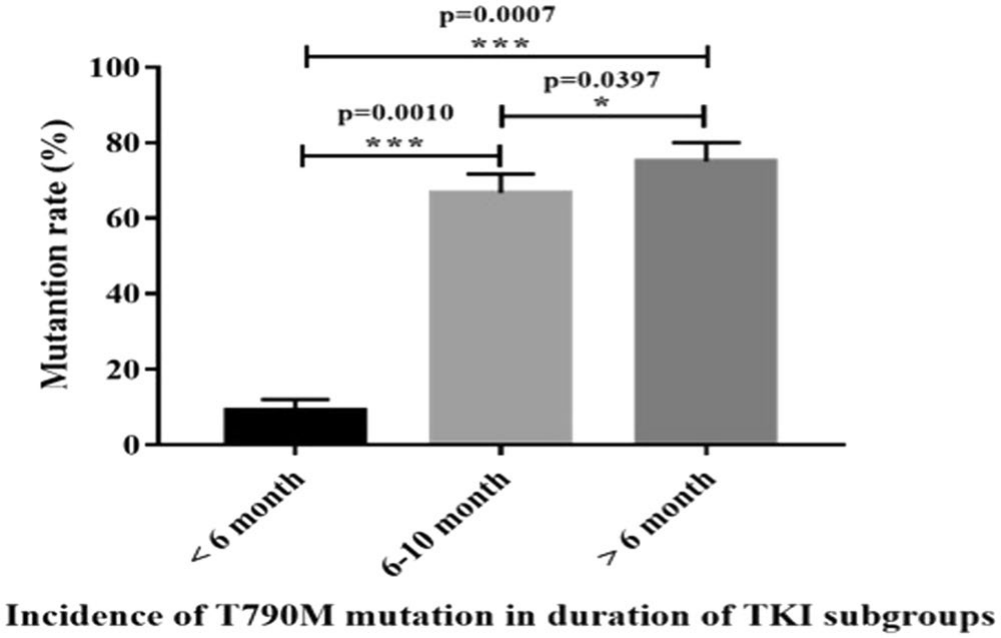
The relationship between the using of first generation TKIs’ time and the T790M mutation.

Among 212 samples, 35 samples were selected to receive first generation TKI. 11 samples’ duration of TKI was less than 6 months, and the incidence of T790M mutation rate was 9.0% (1/11); 12 samples’ duration of TKI was from 6 to 10 months, and the incidence of T790M mutation rate was 66.6% (8/12); 12 samples’ duration of TKI was more than 10 months, and the incidence of T790M mutation rate was 75.0% (9/12). The difference of T790M mutation rate was analyzed according to the duration of TKI. It can be observed that TKI time for 6 to 10 months (*p* < 0.01), and TKI time >10 months (*p* < 0.01) were associated with a greater likelihood of the T790M mutation (Fig. 1).

## Discussion

The EGFR-TKIs have more specific clinical efficacy, compared with standard chemotherapy^19^, when given as second-line or third-line therapy for advanced NSCLC. However, it has been demonstrated that 60% of the patients who received TKI treatment for 6 to 10 months would eventually develop resistance to TKIs. Drug resistance was classified into primary and secondary resistance (acquired resistance). Usually patients who received TKIs for approximately 2 weeks would show an improvement of clinical symptoms, such as tumor mass reduced in radiography and, effect evaluation, including complete remission (CR), partial remission (PR), or stable disease (SD) at a certain time point. If tumors show no apparent response to TKIs during initial treatment, it is called primary resistance. Secondary resistance (acquired resistance) is quite different in definition; an apparently beneficial treatment effect can be observed after starting to take TKIs, but after 6 to 10 months of treatment, the tumor can no longer be suppressed, and may even increase in size, resulting in no improvement in treatment. Currently, many mechanisms of resistance to TKIs have been identified, but mechanisms of primary resistance are not clear. The best described mechanism of primary resistance is a mutation in the KRAS oncogene which is present in 20 to 30% of lung cancer patients. The major mechanism of acquired resistance was reported to be the T790M mutation on exon 20 on the EGFR gene^20,21^. Other mechanisms include amplification of the MET gene^20,21^, PIK3CA mutation^21,22^, BRAF mutation^23^, epithelial-to-mesenchymal transition (EMT)^21^ and small cell lung cancer (SCLC) transformation^20,21^.

It has been reported by two groups that a secondary threonine-to-methionine mutation at codon 790 (T790M) of the EGFR gene is related to acquired resistance to gefitinib and erlotinib (first generation TKIs)^24,25^. Crystal structure modeling has shown that residue T790 is located in the ATP-binding pocket of the catalytic region of EGFR, and it seems to be critical for the binding of erlotinib and gefitinib^24^. Substitution of the threonine at codon 790 with a bulkier residue, such as methionine, would result in steric hindrance to the binding of these two drugs. A secondary T790M mutation has been identified in one tumor^24^ and in three of six tumors^25^ with acquired resistance to gefitinib. In our study, we found the primary mutation rate of T790M was 1.6% (3/177), and the secondary mutation rate was 42.8% (15/35). This change further supports that T790M mutation can be an important player during the development of acquired resistance.

Detection methods for T790M mutations included direct sequencing (DS), amplification refractory mutation system (ARMS), real time quantitative PCR (qPCR), droplet digital PCR (ddPCR), and next generation sequencing (NGS). For the detection of T790M, these detection methods have their own advantages and disadvantages: 1. Compared with ddPCR and NGS, ARMS and Super-ARMS’s detection process is relatively simple and fast, and the cost is lower, but the sensitivity is slightly lower. 2. Compared with ARMS and Super-ARMS, the sensitivity of the ddPCR and NGS is slightly higher, but the detection process is complex and the cost is higher. Direct sequencing is a standard method for the detection of T790M mutations in China, but the process is tedious, time-consuming and the sensitivity is lower^26^. Most of the mutations are somatic mutations, mutant cells which are usually mixed with wild type cells; thus, the extracted DNA often has a large amount of wild type DNA, so the detection of mutation on somatic cells needs higher specificity. At present, direct sequencing is limited and cannot meet clinical needs. The Super ARMS, which we utilized in this research, is an updated technology based on traditional ARMS. We added a self-ringed structure on the Super ARMS primer that can be opened and bounded with a target sequence during the annealing step, to achieve the detection of a certain mutation. This structure strengthened the distinguishing capacity of the primer and thus improved the specificity and sensitivity of the assay. In our study, the Super ARMS’s sensitivity, specificity, PPV, NPV and accuracy were 100.0%, 99.4%, 94.7%, 100.0% and 99.5%, respectively. Its sensitivity was higher than traditional ARMS (48.2–63.6)^26,27^, and other parameters were basically consistent with previous studies^26,27^. There are two advantages of Super ARMS compared with ARMS. First, Super ARMS’s detection sensitivity has been greatly improved (0.2% versus ARMS sensitivity of 1%), allowing for more patients with EGFR mutations to be detected. Second, compared with ARMS, Super ARMS is especially suitable for plasma samples because of its higher sensitivity. Digital PCR (dPCR) is a highly sensitive gene mutation detection method that is based on the compartmentalization and amplification of single DNA molecules. Quantification of compartments with endpoint fluorescence after the PCR process reveals the number of copies of target DNA. Droplet digital PCR (ddPCR) is one such dPCR technology that is based on the compartmentalization of DNA into droplets. In ddPCR, individual DNA fragments are compartmentalized into more than a million droplets, which are then amplified in parallel. ddPCR is a promising technology as its speed, cost, and ease of use are similar to other PCR-based assays, yet the sensitivity and quantitative nature of this assay offers broader clinical applications. Currently, ddPCR has been proved to be an efficient and reliable method for detection of the T790M mutation^28,29^. Therefore, all samples by Super ARMS were verified by ddPCR again in our study. In particular, one sample was judged as a false positive when, compared with ddPCR result. Two reasons may account for this issue. First, the detection of the result was close to the critical value, and then it was misjudged as positive. Additionally, PCR contamination can also lead to false positive. Despite one false positive case, the technique can be further improved and intensified.

The EGFR mutation rate was 30.1% (64/212) among patients with NSCLC, which was in the range of previous studies (30–50%)^3,30–32^. Similarly, female, never-smokers, brain metastasis and adenocarcinoma were associated with a higher rate of EGFR mutations in this study. Partly, we were just investigated the prevalence of the EGFR-T790M mutations in Yunnan province of southwest China.

The T790M mutation rate was 8.4% among patients with NSCLC, which was in the range of previous reports^33,34^. In the non-TKI plasma samples, female, never-smokers and adenocarcinoma were not significantly relationship with a higher T790M mutation rates in NSCLC patients in this study. This was similar with other previous studies^34,35^. Brain metastasis was correlated with a higher T790M mutation rate in NSCLC patients. This was also similar with other reports^34,35^. However, the T790M mutation rate was 0.0% (0/31) in TNM stage (Ia-IIIa). Specifically, it was lower than the T790M mutation rate in TNM stage (IIIb-IV), which was 9.9% (18/181); no significant association was found in TNM stage. Although this does not match other previous studies, its tendency was pronounced^36^. There are three explanations may better account for this dispute. First, we only collected available data in this study, which may lead to a selection bias. Second, the number of samples were not enough to reflect the relationship between the T790M mutations and TNM stage of NSCLC in Yunnan province. Finally, biopsy is still the gold standard for detecting T790M mutations. However, since plasma was used to detect the mutation of T790M in this study, it may also lead to bias. In the post-TKI plasma samples, female, never-smokers and adenocarcinoma were not significantly associated with the higher T790M mutation rate in NSCLC patients in this study. This was similar with other previous studies^34^. Brain metastasis was correlated with a higher rate of T790M mutations, which was in good agreement with other reports^35^. The T790M mutation rate was 42.8% (15/35) in TNM stage (IIIb-IV). Therefore, regardless of TKI treatment status, brain metastasis was correlated with a higher rate of NSCLC patients.

In the non-TKI plasma samples, the T790M mutation rate was 1.6% (3/177). This was lower than in other previous studies that used ARMS detective method^35,37^. Therefore, both Super AMRS and ARMS were limited for the detection of primary T790M mutation. In the post-TKI plasma samples, the T790M mutation rate was 42.8%, which was consistent with other previous reports^35,37^ and much higher than the non-TKI plasma samples (*p* < 0.001). To further prove that the main mechanism of acquired resistance is secondary T790M mutation. We also analyzed the relationship between T790M mutation rate and duration of TKI to explore the mechanism of acquired resistance. We found that duration of TKI for 6 to 10 months (*p* < 0.01) and >10 months (*p* = 0.01) were associated with a higher T790M mutation rate. It was also confirmed that many patients who chose to use TKI for at least 6 months would develop resistance, and 60–70% of TKI resistance was related to the T790M mutation^38^. Similarly, the T790M mutation rate was 66.6% (8/12) in duration of TKI (6–10 months). This was lower than the duration of TKI (>10 months), 75% (9/12), which was consistent with other reports^37,38^.

Our previous studies found the rate of EGFR mutations in Xuanwei City was different from that of general populations^3^, which motivated our exploration on whether the T790M mutation has regional specificity in Xuanwu City. However, we found that NSCLC patients in Xuanwei City had a lower T790M mutation rate compared with non-Xuanwei City in our study. In addition, no significant association was found in Xuanwei origin. Therefore, other factors may play an important role in the progression and development of lung cancer except genetic factors in Xuanwu City. Since our study recruited patients in a single center and the number of Xuanwei NSCLC patients’ samples were not large enough, which is lead the fact that our sample could not better reflect the rate EGFR-T790M mutations in Xuanwei City. But, further studies are expected.

Patients with EGFR-mutant NSCLC derive significant therapeutic benefit from TKIs. Unfortunately, acquired resistance is an inevitable consequence of this treatment strategy. Consequently, it is particularly important to find a treatment strategy that will overcome resistance of TKIs resulting from the T790M mutation. The treatment strategy for drug resistance at the present stage are as follows: 1. Delay acquired resistance to TKIs: Evaluation of patients with progression on first-line TKIs therapy is mainly dependent on RECIST (Response Evaluation Criteria in Solid Tumors), but this does not provide guidelines for drug withdrawal. Some patients may have RECIST progression based on tumor measurements but show continued clinical benefit from therapy. Many asymptomatic patients can delay a change in therapy for 2–3 months^39^. Two studies reported that some NSCLC patients were sensitive to EGFR inhibitors, and when the TKIs were interrupted that would lead cancer progression to accelerated^40,41^. As a result, it is still beneficial to continue to take TKIs for many patients, even after TKI resistance develops. However, it must be clear when the TKIs should be stopped: (1) New metastatic lesions (brain metastasis not included, because of the blood-brain barrier), especially the emergence of a wide range of metastases. (2) Disease related symptoms increased significantly. (3) The tumor grows rapidly. 2. Second-generation TKIs: Afatinib is a dual EGFR/HER2 inhibitor that is now FDA-approved for the first-line treatment of lung cancers with EGFR L858R mutations or exon 19 deletions. NCCN also suggested using afatinib to replace first generation TKIs after they induce drug resistance^42^. 3. Third-generation TKIs: The 2016 edition of the NCCN, NSCLC clinical practice guidelines recommended tagrisso (AZD9291) as a second-line treatment for patients who have the T790M mutation or have received first generation TKI that led to drug resistance^15^. 4. MET inhibition: MET amplification is detected in approximately 5% of tumors with acquired resistance to TKIs^21^. Therefore, therapies targeting MET may be an effective strategy in MET-amplified tumors^43^. 5. Immune therapy: According to a multicenter clinical study, the first generation of TKI + PD-1 monoclonal antibody can reach 19% RR and PFS in 24 weeks^44^.

Overall, it is feasible to detect tumor-derived T790M mutations in the EGFR gene using cfDNA from patients with NSCLC using Super ARMS. The rate of the T790M mutations in NSCLC patients of Xuanwei City was showed no significant difference to the other Asian populations. Our studies showed that NSCLC patients with brain metastasis, history of first generation TKIs and duration of TKIs > 6 months, were relationship with a higher T790M mutation rate. In addition, Super ARMS was used to detect of T790M mutation rate, which is one of the most important recent breakthroughs in NSCLC oncology. Considering the limitation of our study, further research should explore a large sample size from multiple-centers in Yunnan Province to make it more representative for the overall population. Through analysis of significance and clinical productivity of detection technology for the T790M mutation, we found that the detection technology for the T790M mutation could help patients to decide on a strategy of treatment, help them save treatment cost and obtain a better prognosis, and perhaps most importantly, provide meaningful guidance for treatment of drug resistance in patients with the T790M gene mutation.

## Materials and Methods

### Study Population

We selected patients with NSCLC undergoing routine tumor genotyping in the Third Affiliated Hospital of Kunming Medical University between November 2015 and June 2016 as our primary study population. Written informed consent was obtained from all included individuals. The study was approved by the ethics committee of the Third Affiliated Hospital of Kunming Medical University, and complied with the Declaration of Helsinki and Good Clinical practice guidelines.

Eligibility criteria were: (1) adults (>18 year) who were residents of Yunnan province; (2) histology or cytology confirmed NSCLC; (3) patients who have received the first generation of TKIs were selected first and patients with newly diagnosed or postoperative recurrence were selected second.

#### Exclusion criteria were: Any specimen that might be contaminated was excluded

In our study, specimens from 266 patients’ who were diagnosed with NSCLC were collected. For some reasons (such as insufficient sample size, substandard quality or some patients with incomplete information), only 212 patients’ specimens were enrolled in this research. The patient data were subgrouped according to age, sex, stage, and treatment information, such as whether the patients were receiving EGFR-TKI treatment, whether they had received chemotherapy or whether they had received other treatments.

#### Plasma isolation and DNA Extraction

Patients’ blood samples were collected with a tube for EDTA anticoagulant (10 mL) and centrifuged at 2, 000 g and 8, 000 g for 10 min at 4 °C within 2 hours of collection. The plasma supernatant was isolated, and DNA was extracted immediately. DNA from the plasma supernatant samples was extracted with a Circulating DNA Kit (AmoyDx, Xiamen, China) according to the manufacturer’s instructions^45^.

#### EGFR-T790M detection

T790M status in plasma supernatant samples was determined by using the Super ARMS T790M Mutation Detection kit (AmoyDx, Xiamen, China)^46^.

According to the ratio of 0.4 μL P-T790M Enzyme Mix, 60 μL P-T790M Reaction MixA and 5 μL P-T790M Reaction MixB per sample, we transferred the appropriate amount of P-T790M Enzyme Mix and P-T790M Reaction MixA/B into a sterile tube. And then transferred 65.4 μL of the above mixed solution into the appropriate PCR tubes. 15 μL sample DNA, P-T790M positive control (PC) or 15 μL ddH_2_O (no-template control, NTC) was added to the appropriate PCR tubes. The PCR tubes were then placed into the real-time PCR instrument.

Real-time PCR was carried out using the cycling conditions described in Table 6.

1. For P-T790M Positive Control, the Ct value for both FAM and HEX/VIC signal should be less than 20, but variation may occur due to different threshold settings on different instruments.
2. Make sure that each sample gives a HEX/VIC signal and the Ct value should be less than 19. If the Ct value of HEX/VIC signal ≥19, it shows that the DNA sample contains PCR inhibitors or the DNA amount is insufficient, indicating that the DNA needs to be re-extracted, and the whole experiment should be carried out again.
3. Check the FAM Ct value for each sample.

**Table 6:** Cycling parameters.

Stage	Temperature	Time	Cycles
1	95 °C	10 min	1
2	95 °C	40 s	15
	64 °C	40 s	
	72 °C	30 s	
3	93 °C	40 s	28
	60 °C	45 s	
	72 °C	30 s	

Sample Data Analysis.

If the FAM signal has positive amplification, and the ΔCt value is <8, the sample is classified as EGFR T790M positive. If the ΔCt value is ≥8, the sample is classified as negative or below the detection limit of the kit.

The calculation of ΔCt: ΔCt = FAM Ct value − HEX/VIC Ct value. The FAM Ct value indicates the Ct value of the sample’s FAM signal; the HEX/VIC Ct value indicates the Ct value of the sample’s HEX/VIC signal.

### Droplet Digital PCR verification

Each Super ARMS sample was verified again by digital PCR. Droplet Digital PCR was used verified the accuracy of the results analysis performed by the QX200 system. Samples are partitioned into 20000 droplets by a droplet generator, and droplets are amplified by PCR. Thermal cycling profile for T790M assay was as follows: 10-min incubation at 95 °C followed by 45 cycles of 95 °C for 15 sec and, 60 °C for 60 sec and then maintained at 4 °C. After PCR, the 96-well PCR plate was loaded on the droplet reader to read the droplets from each well of the plate. Quanta Soft software was used in the analysis of the ddPCR date for allele calling. Single droplets occasionally showed up as random events when NTC reactions were tested. Therefore, the samples that had at least 2 droplets in the positive area for FAM signal were counted as positive for the mutation^47^.

### Calculation of detection efficiency

Sensitivity (true positive rate) = (A/A + C) × 100%; Specificity (true negative rate) = (D/B + D) × 100%; Positive predictive value (PPV) = (A/A + B) × 100%; Negative predictive value (NPV) = (D/C + D) × 100%; accuracy(diagnostic efficiency) = (A + D/A + B + C + D) × 100% (A represents the number of patient detected as positive by both methods; B and C represent number of patients who were discrepant between the two methods; D the number of patients detected as negative by both methods).

### Statistical analysis

Pearson Chi-square was used to analyze the relationship between T790M mutations and clinical factors (such as age, sex, smoking status, histological type, tumor site, source of NSCLC patients, whether they received EGFR-TKIs or not, occupation, *et al*.). Super ARMS’ sensitivity, specificity, positive predictive value, negative predictive value and accuracy were also calculated according to statistical data. All the statistics were performed by software of SPSS 22.0 (SPSS Inc., Chicago, IL, USA) *p* < 0.05 was considered to be statistically significant.
